# A Pharmacodynamic Study of Aminoglycosides against Pathogenic *E. coli* through Monte Carlo Simulation

**DOI:** 10.3390/ph17010027

**Published:** 2023-12-24

**Authors:** Eon-Bee Lee, Kyubae Lee

**Affiliations:** 1 Laboratory of Veterinary Pharmacokinetics and Pharmacodynamics, College of Veterinary Medicine, Kyungpook National University, Daegu 41566, Republic of Korea; eonbee@gmail.com; 2 Department of Medical Engineering, Yonsei University College of Medicine, 50-1 Yonsei-ro, Seoul 03722, Republic of Korea

**Keywords:** pharmacodynamic function, antimicrobial resistance, *E. coli*, aminoglycoside, Monte Carlo simulation

## Abstract

This research focuses on combating the increasing problem of antimicrobial resistance, especially in *Escherichia coli* (*E. coli*), by assessing the efficacy of aminoglycosides. The study specifically addresses the challenge of developing new therapeutic approaches by integrating experimental data with mathematical modeling to better understand the action of aminoglycosides. It involves testing various antibiotics like streptomycin (SMN), kanamycin (KMN), gentamicin (GMN), tobramycin (TMN), and amikacin (AKN) against the O157:H7 strain of *E. coli*. The study employs a pharmacodynamics (PD) model to analyze how different antibiotic concentrations affect bacterial growth, utilizing minimum inhibitory concentration (MIC) to gauge the effective bactericidal levels of the antibiotics. The study’s approach involved transforming bacterial growth rates, as obtained from time–kill curve data, into logarithmic values. A model was then developed to correlate these log-transformed values with their respective responses. To generate additional data points, each value was systematically increased by an increment of 0.1. To simulate real-world variability and randomness in the data, a Gaussian scatter model, characterized by parameters like κ and EC_50_, was employed. The mathematical modeling was pivotal in uncovering the bactericidal properties of these antibiotics, indicating different PD MIC (zMIC) values for each (SMN: 1.22; KMN: 0.89; GMN: 0.21; TMN: 0.32; AKN: 0.13), which aligned with MIC values obtained through microdilution methods. This innovative blend of experimental and mathematical approaches in the study marks a significant advancement in formulating strategies to combat the growing threat of antimicrobial-resistant *E. coli*, offering a novel pathway to understand and tackle antimicrobial resistance more effectively.

## 1. Introduction

Over the past few decades, antimicrobial resistance has emerged as one of the most pressing health concerns worldwide [1]. Central to this concern is the resistance exhibited by common pathogens, with *Escherichia coli* (*E. coli*) being a prime example [2]. This resistance is not merely an academic interest; it poses real-world consequences. As these microorganisms evolve and develop resistance, they render many previously effective therapeutic agents obsolete [3]. The resulting dearth in the therapeutic strategies complicates clinical treatments and prolongs patient recovery [4].

Within this backdrop, aminoglycosides have stood out as a beacon of hope [5]. As a potent class of antibiotics, they have been tailored specifically to counter Gram-negative bacterial threats, a category to which *E. coli* belongs [5]. Aminoglycosides enter bacterial cells passively, then actively cross the inner membrane, where they hinder protein synthesis by binding to the 30S ribosomal subunit, leading to defective proteins and bacterial cell death [6]. This disruption in protein synthesis is the primary mode of their bactericidal action [7]. However, the efficacy of antibiotics is not solely contingent on their direct bacterial action but also on a complex dance of absorption, distribution, metabolism, and excretion in the human body, collectively referred to as pharmacokinetics (PK) [8]. Moreover, it is not just about how the body processes these drugs; it is equally about how these drugs, once administered, influence both the pathogen and the host. This sphere of influence, known as pharmacodynamics (PD), encapsulates the drug’s therapeutic and adverse effects [9]. The relationship between PK and PD is integral for determining the dosage regimen of a drug [10], optimizing its therapeutic efficacy [11], and minimizing adverse effects [12].

Despite the pivotal role that aminoglycosides play, especially within the PK/PD framework, there exists a puzzling gap. Comprehensive and granular data on these antibiotics, spanning from their absorption kinetics to their bacterial eradication rates [13,14], is not as abundant as one would expect. This paucity of data is especially surprising given the gravity of the antimicrobial resistance issue and the prominence of aminoglycosides in counteracting such resistance [15]. It underscores an urgent need for research endeavors to inform more robust clinical strategies. The E_max_ model, which holds significant importance in PK/PD for quantifying the effect of a drug in relation to its concentration, is intricate due to its reliance on in vivo studies [16]. These in vivo studies often introduce complexities due to physiological variables, making the extrapolation of results challenging [17]. Recognizing this, previous study pioneered an alternative approach through their PD modeling [18]. While conceptually parallel to the E_max_ model, it leverages mathematical computations, offering a more systematic, replicable, and less labor-intensive method.

Driven by the above gaps and innovations, our research embarked on a dual-phase journey. The first phase involved in vitro time–kill assays of five distinct aminoglycosides, gauging their efficacy against *E. coli*. These assays, through controlled conditions, aimed to chart the bactericidal trajectory of each aminoglycoside over time. Armed with this data, the subsequent phase employed the PD model proposed by previous research [18]. This study aimed to enhance understanding of aminoglycosides’ potential against increasing antimicrobial resistance by connecting experimental results with mathematical models. Through detailed computational analysis, we seek to offer dependable methods that assist in making well-informed decisions for therapeutic strategies.

## 2. Results

### 2.1. MIC and MBC

**Figure 1:**
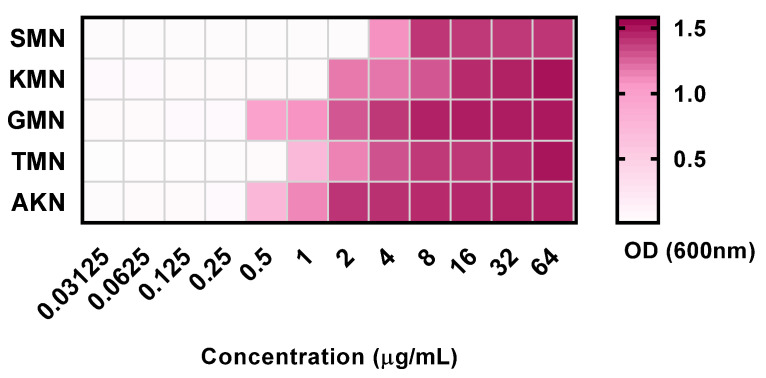
Optical density at 600 nm of 5 aminoglycoside antibiotics with concentration variations between 0.03125 to 64 μg/mL. Streptomycin, SMN; kanamycin, KMN; gentamicin, GMN; tobramycin, TMN; amikacin, AKN.

In the assessment of five antibiotics against *E. coli*, the findings revealed the following MIC and MBC values (Figure 1 and Table 1). For SMN, the MIC was determined to be 2, with an MBC of 4 and an MBC-to-MIC ratio of 2. KMN exhibited an MIC of 1, an MBC of 2, and a ratio of 2. GMN had an MIC of 0.25, an MBC of 1, and a ratio of 4. TMN presented with an MIC of 0.5, an MBC of 1, and a ratio of 2. Lastly, AKN demonstrated an MIC of 0.25, an MBC of 1, and a ratio of 4. These values provided insights into the efficacy of each antibiotic in inhibiting and killing *E. coli*.

### 2.2. Time–Kill Curves against E. coli

**Figure 2:**
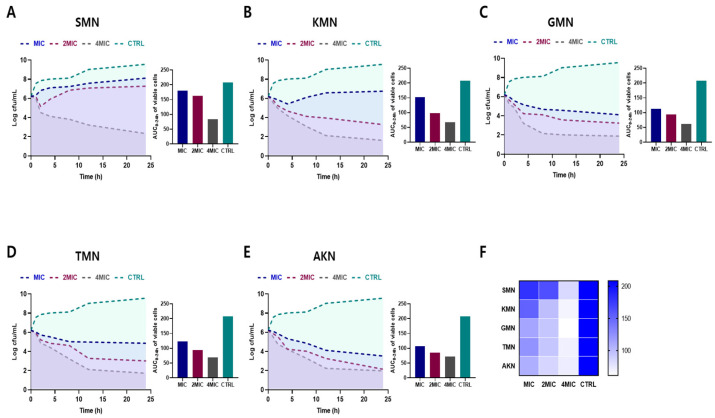
Time–kill curves and area under the curve (AUC) of viable cells against *E. coli* ATCC 43888. Bacterial count measurements of various antibiotics (SMN (**A**), KMN (**B**), GMN (**C**), TMN (**D**), AKN (**E**)) with various concentration (MIC, 2MIC and 4MIC) against *E. coli* for 24 h. (**F**) Comparative visualization of antibiotic effectiveness using AUC of viable cells. Color gradients in heat map symbolize the range of activity. Streptomycin, SMN; kanamycin, KMN; gentamicin, GMN; tobramycin, TMN; amikacin, AKN.

Figure 2A–E illustrates the bacterial count measurements for several antibiotics against *E. coli* over a 24 h period. At the onset (0 h), all antibiotics exhibited consistent bacterial counts across all concentrations (MIC, 2MIC, and 4MIC), as well as the control, with a value of 6.21 Log cfu/mL. As time progressed, the control samples consistently showed an upward trajectory in bacterial growth, culminating at 9.56 at the 24 h mark. In contrast, for all antibiotics, as the concentration increased, bacterial counts typically decreased. By 24 h, SMN’s bacterial count was highest at MIC with 8.12 Log cfu/mL, but dwindled to 2.32 Log cfu/mL at 4MIC. Similarly, KMN displayed a count of 6.75 Log cfu/mL at MIC and plummeted to 1.61 Log cfu/mL at 4MIC. GMN, TMN, and AKN followed the same trend. This overarching pattern underscored the potent growth-inhibitory effects of these antibiotics on *E. coli*, with their efficacy generally amplifying at higher concentrations.

The area under the curve (AUC) of viable cells, which were consistent with time–kill curves, offered insights into the performance of various antibiotics against *E. coli* over time. Heat map results, as shown in Figure 2F, also provided a visual representation of the comparative efficacy of antibiotics. Darker shades indicated reduced efficacy activity, while lighter shades suggested higher activity. For SMN, the AUC values are 179.3, 162.5, and 83.22 for MIC, 2MIC, and 4MIC concentrations, respectively, while the control showed a higher AUC of 208.5. KMN demonstrated AUC values of 151.8, 98.47, and 67.5 for MIC, 2MIC, and 4MIC respectively, again with a control AUC of 208.5. GMN has AUC measurements of 112.9, 93.77, and 61.38 for its respective concentrations. TMN posted AUC results of 123.3, 94.17, and 69.63, while AKN registered values of 107.5, 85.08, and 71.29. This findings indicated the effectiveness of the antibiotics in inhibiting bacterial growth over time, with lower AUC values representing better antibiotic efficacy.

### 2.3. PD Modeling through Simulation

**Figure 3:**
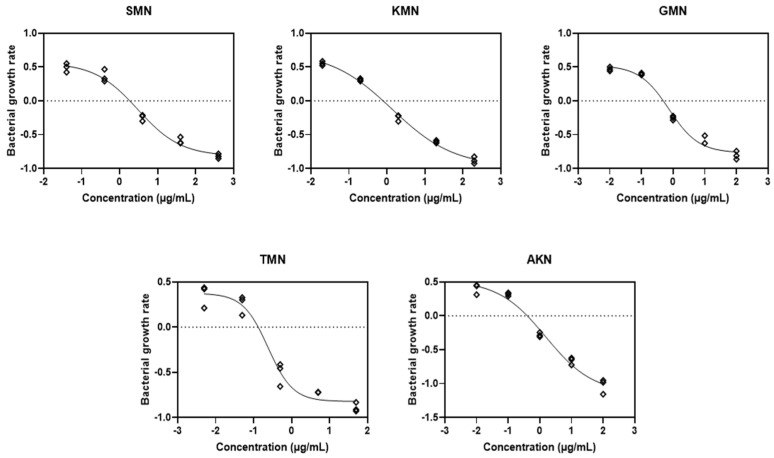
Fitting the pharmacodynamic model to the time–kill curves. Streptomycin, SMN; kanamycin, KMN; gentamicin, GMN; tobramycin, TMN; amikacin, AKN.

Equation (3), which represented the PD function, corresponded to the observed net growth rates of bacteria, as depicted in Figure 2A–E. A model based on logarithm versus response was developed from this. Consequently, this approach facilitated the derivation of four key parameters: ψ_max_, ψ_min_, κ, and EC_50_, which are elaborately presented in Figure 3 and detailed in Table 2.

Based on the parameter estimates displayed in Table 2 for various aminoglycosides, we can observe distinct PD profiles for each antibiotic. For SMN, the ψ_max_ was estimated at 0.5651 with a confidence interval (CI) ranging from 0.4419 to 0.7845. Conversely, the ψ_min_ showed a significant inhibitory effect at −0.8166 (CI: −1.028 to −0.6976). The Hill coefficient, indicative of the steepness of the drug effect curve, was noted at −0.7631 (CI: −1.244 to −0.4600), and the EC_50_ value, the concentration required to achieve half the maximal antibacterial effect, was 2.996 (CI: 1.781 to 5.250). This antibiotic also showed a high R^2^ value of 0.9842, suggesting a strong fit to the observed data. KMN, on the other hand, presented a higher ψ_max_ of 0.7290 (CI: 0.5889 to 1.022) indicating a faster growth rate of bacteria in the absence of the drug. Its ψ_min_ was −0.9728 (CI: −1.213 to −0.8488), and the Hill coefficient was −0.5324 (CI: −0.7153 to −0.3553), suggesting a less steep response curve than SMN. The EC_50_ value for KMN was lower at 1.374 (CI: 0.8118 to 2.202), and the R^2^ value was exceptionally high at 0.9938, denoting a very accurate model fit. Each antibiotic’s individual PD parameters, including GMN, TMN, and AKN, similarly reflect their unique inhibitory profiles and potency, with varying degrees of bacterial growth inhibition and death rates, as evidenced by their respective EC_50_ values and Hill coefficients.

**Figure 4:**
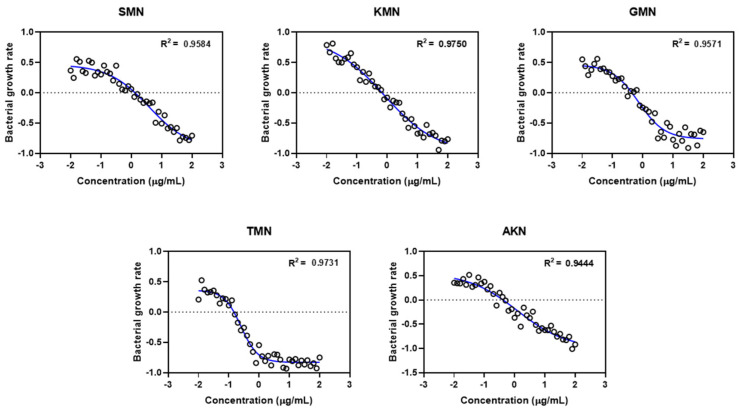
Gaussian scatter model via pharmacodynamic functions for five different antimicrobials. Streptomycin, SMN; kanamycin, KMN; gentamicin, GMN; tobramycin, TMN; amikacin, AKN.

**Figure 5:**
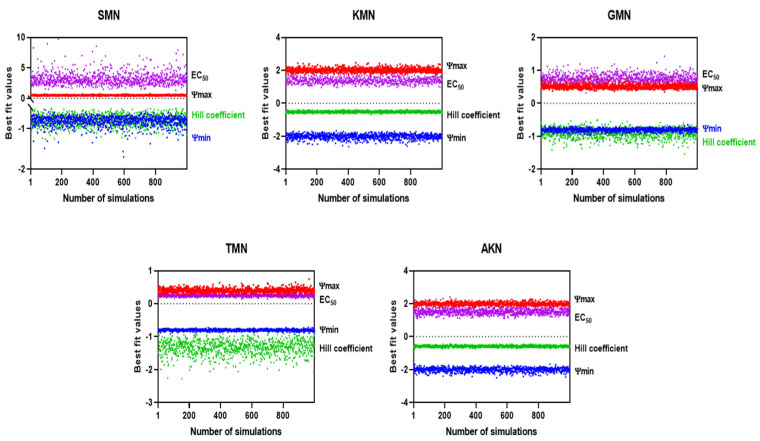
Simulated EC_50_ and Hill coefficient by Monte Carlo method.

These calculated values then facilitated the introduction of the Gaussian scatter model, followed by the application of the Monte Carlo simulation. The PD function provided an excellent fit for all five antibiotics, as evidenced by the adjusted R^2^ values shown in Figure 4. Simulated ψ_max_, ψ_min,_ EC_50_, and Hill coefficient were described in Figure 5. Table 3 presented the parameter estimates for various antibiotics determined through Monte Carlo simulations, with a particular emphasis on the zMIC values. SMN has a zMIC of 1.22, indicating the concentration at which it inhibits the growth of the bacterial population. KMN exhibits a lower zMIC value of 0.89 ± 0.52, suggesting a potent antibacterial effect at lower concentrations. GMN shows an even lower zMIC of 0.21 ± 0.02, highlighting its strong efficacy in inhibiting bacterial growth.

TMN presents a zMIC of 0.32 ± 0.15, which is comparable to GMN, indicating its effectiveness in halting bacterial proliferation. AKN has the lowest zMIC of 0.13 ± 0.02 among the group, suggesting that it is the most effective at inhibiting bacterial growth at minimal concentrations. These zMIC values provide critical insights into the pharmacodynamics of these antibiotics, showcasing their potential effectiveness at specific concentrations against bacterial growth.

## 3. Discussion

Addressing the challenge of antimicrobial resistance in *E. coli* necessitates the development of rigorous and consistent methods for the in vitro evaluation of antimicrobial interventions [2]. The research presented in this study performed an in vitro time–kill assay of aminoglycosides against *E. coli*. By using this assay, we sought to gain a deeper understanding of the dynamic interaction between antimicrobials and bacterial populations over time, which is essential for predicting treatment outcomes in clinical settings. The incorporation of the PD model, which elucidates the intricate relationship between antimicrobial concentration and bacterial growth rate, added another layer of sophistication to our analysis. Such models are paramount in bridging the gap between in vitro results and their clinical implications. They offer insights into the optimum concentration levels needed to curb bacterial growth, thereby facilitating a more targeted approach to dosing regimens.

The MBC to MIC ratio serves as a critical parameter in understanding the bactericidal nature of antibiotics [19]. It is widely accepted that an antibiotic with an MBC/MIC ratio of 4 or less is generally considered bactericidal against a particular microorganism [20]. In our study, evaluating five antibiotics against pathogenic *E. coli*, the findings suggested promising results in terms of bactericidal activity. Specifically, all the antibiotics tested exhibited an MBC/MIC ratio of 4 or less, classifying them as bactericidal agents against this strain of *E. coli*. SMN, KMN, and TMN each displayed a ratio of 2, which indicated a strong bactericidal potential since their killing concentrations are only twice their inhibitory concentrations. On the other hand, GMN and AKN presented with a ratio at the threshold of 4. While still within the bactericidal range, this suggested that their bactericidal concentrations are four times their inhibitory concentrations, marking a relatively higher distinction between inhibition and killing capabilities when compared to the other antibiotics.

The AUC derived from the time–kill assay is an indispensable metric when assessing the efficacy of antimicrobials [21]. Essentially, it offers a quantitative representation of bacterial response over time when subjected to antibiotic treatment. In the context of antimicrobial susceptibility, a smaller AUC typically denotes a more potent antibacterial effect as it indicates fewer viable bacterial cells over the assay’s duration. Delving into specifics, the AUC values for SMN across various concentrations (MIC, 2MIC, and 4MIC) clearly indicated a concentration-dependent effect. As the concentration increased, the AUC diminished, underscoring a heightened antibiotic effect. A similar trend is discernible for KMN, with its AUC values diminishing progressively with increasing antibiotic concentration.

The PD function served as a tool to elucidate the relationship between bacterial vitality rates and varying concentrations of antibiotics belonging to different classes [18]. This function corresponds closely with E_max_ models previously mentioned in other report [22]. Within this function, there are four essential parameters clearly outlined. Firstly, ‘ψ_max_’ highlights the peak bacterial growth rate when no antibiotic is present. ‘ψ_min_’ depicts the lowest net bacterial growth rate when confronted with high antibiotic concentrations. ‘zMIC’ acts as an indicator for the PD MIC. The Hill coefficient underscores the sensitivity of bacterial growth or mortality rates to changes in antibiotic concentrations [23]. Central to this is the Hill coefficient, a pivotal determinant of the curve’s gradient, especially around the zMIC point. This coefficient provides profound insights into how alterations in antimicrobial concentrations influence bacterial elimination [24]. Intriguingly, previous research established a noteworthy correlation: antimicrobials with a concentration-dependent manner, epitomized by drugs like ciprofloxacin, typically align with elevated Hill coefficients [18]. In contrast, time-dependent antimicrobials like tetracycline tend to have lower Hill coefficients. An in-depth analysis of Table 3, which details the parameter estimates obtained via Monte Carlo methods, further supported and enhanced these results. The data show GMN having a Hill coefficient of 1.00 ± 0.06 and TMN with a coefficient of 1.56 ± 0.20, hinting at a likely concentration-dependent mechanism. In contrast, AKN, with a Hill coefficient of 0.53 ± 0.14, appears to indicate a tendency towards a time-dependent mode of action. While AKN is generally recognized as concentration-dependent [25], previous research has revealed its time-dependent toxic effects on the renal functions of male Wistar rats [26]. The research found variations in toxic effects, like decreased creatinine clearance and urinary excretion of furosemide, based on the timing of AKN administration. The MICs of these antibiotics were assessed in vitro using a twofold dilution method. The zMICs were found to align with the range determined by the dilution method. However, greater precision is offered by them, as they are not constrained by a twofold dilution method. The parameter estimates from Table 3, obtained through Monte Carlo simulations, revealed distinct pharmacodynamic profiles of various antibiotics against *E. coli*. Antibiotics like SMN and KMN show significant differences in their ψmax (0.46 ± 0.05, 0.90 ± 0.13) and ψ_min_ values (−0.92 ± 0.13, −0.99 ± 0.12), indicating varied ranges of action and potencies. SMN showed intricate interactions with bacterial cells, whereas KMN revealed a stable and strong efficacy over various concentrations. This was further explored in the study of streptomycin resistance in *E. coli* mutants [27]. GMN, known for its pronounced concentration-dependent impact, a finding corroborated by earlier studies on intracellular Yersinia pestis [28], stands in stark contrast to TMN. The latter displays a distinct mode of action, as evidenced by its notably high negative Hill coefficient, the most extreme among the antibiotics evaluated in the study. AKN stood out with a potential time-dependent action, suggested by its Hill coefficient and the lowest zMIC value. These findings underscore the diverse mechanisms of action of these antibiotics, crucial for understanding their effectiveness against antimicrobial-resistant *E. coli* strains.

The obtained parameters from in vitro time–kill curves, notably the ψ_max_, ψ_min_, and zMIC in tandem with the Hill coefficient, crafted the PD profile of these antibiotics. Tailoring antibiotic therapy based on such insights could pave the way for a reproducible and affordable strategy to measure the antibiotics’ properties.

The role of PD tackling antibiotic resistance has gained paramount importance. Recent research has enriched our knowledge in this domain, notably in the area of gonorrhoea caused by *Neisseria gonorrhoeae*, where an increasing resistance to conventional treatments has been observed [29]. To address this, a unique in vitro time–kill curve assay was innovatively used, revealing the effectiveness of nine different antimicrobials against established reference strains. The study emphasized the crucial role of this approach, especially through a PD lens, in shaping future gonorrhoea treatments. In another study, the intrinsic qualities of antimicrobial peptides, recognized for their unique PD attributes and resistance to bacteria, were scrutinized [30]. A detailed analysis revealed their effects on *Staphylococcus aureus*, demonstrating an adaptive PD relationship under extended drug exposure, underlining the necessity to comprehend these adaptations to manage resistance development. Another research focused on evaluating the impact of specific antimicrobials on *Neisseria gonorrhoeae*’s growth [31]. Detailed analyses through PD functions were carried out, revealing that higher doses of ceftriaxone might be potent against particular *Neisseria gonorrhoeae* variants and introducing GMN as a potential contender for treatment.

In the study presented, Gaussian models were applied to examine the data distributions and identify primary patterns and anomalies within the data set. Following this, Monte Carlo simulations were executed to predict outcomes, taking into account the stochastic nature and inherent fluctuations of the system being studied. Although Monte Carlo simulations are pivotal for assessing stochastic phenomena, integrating them with Gaussian models introduces certain complexities. One notable limitation is the assumption in the simulation process that variables act independently, an assumption which may not hold true in Gaussian frameworks where variables often exhibit interdependencies that could influence the simulation outcomes. Despite integrating Gaussian models with Monte Carlo simulations, the results of this study showed a linear correlation (as reflected by the R-squared value). Therefore, while the methodologies employed herein have provided valuable insights, cautious interpretation is required. It is recommended that these methods be supplemented with additional analytical techniques to ensure more robust and reliable results. This multifaceted approach would help to mitigate any methodological limitations and provide a more holistic understanding of the data and their implications.

Although the results of this research are encouraging, it is crucial to acknowledge the intrinsic constraints associated with in vitro experiments. Clinical conditions in actual practice are considerably more intricate, shaped by numerous elements such as the host’s immune response, the virulence of the bacteria, and the pharmacokinetics (PK) of the drug. However, the integration of time–kill curve assays and sophisticated PD modeling has laid a robust groundwork for future research endeavors to expand upon.

## 4. Materials and Methods

### 4.1. Chemicals and Reagents

The antibiotics (streptomycin (SMN), kanamycin (KMN), gentamicin (GMN), tobramycin (TMN), and amikacin (AKN)) used in the study were obtained from Sigma-Aldrich (St. Louis, MO, USA). They were prepared for use by dissolving them according to the provided guidelines and recommendations.

### 4.2. Bacteria Culture

*Escherichia coli* (*E. coli*) ATCC 43888 was acquired from the American Type Culture Collection (ATCC). The procured bacteria were cultured on Luria Bertani (LB) agar plates (BD, Diagnostics, Sparks, MD, USA) and allowed to incubate at a temperature of 37 °C for a period of 24 h. After the incubation, emerging colonies were selected and transferred into 5 mL of Mueller Hinton broth (MHB) (BD, Diagnostics, Sparks, MD, USA) and incubated overnight at 37 °C. The bacteria were then subcultured into another 5 mL of the same medium and maintained at 37 °C, with agitation at 180 rpm in a shaker/incubator for a duration of 3 h, facilitating the bacteria to reach the mid-logarithmic growth phase [32].

### 4.3. Minimum Inhibitory Concentration (MIC) and Minimum Bactericidal Concentration (MBC) of Antibiotics against E. coli

The minimum inhibitory concentration (MIC) of antibiotics (SMN, KMN, GMN, TMN, and AKN) against *E. coli* O157:H7 strain ATCC 43888 was assessed using a two-fold serial dilution method, with concentration variations between 0.03125 to 64 μg/mL in accordance with the guidelines of the Clinical and Laboratory Standards Institute (CLSI) [33]. After inoculation, the plates were incubated at 37 °C for 24 h. The MIC was determined as the lowest concentration of antibiotic that visually inhibited bacterial growth in the medium. A microplate reader (Versamax™, Idaho Emmett, ID, USA) was used to confirm the results. For establishing the minimum bactericidal concentration (MBC), samples from three concentrations above the determined MIC, where no visible bacterial growth was observed, were placed onto LB plates. These plates, after being incubated at 37 °C for 24 h, were examined to recognize a 3log10 reduction in the initial bacterial count.

### 4.4. Time–Kill Curves of Antibiotics against E. coli

The in vitro time–kill curves of antibiotics (SMN, KMN, GMN, TMN, and AKN) against *E. coli* O157:H7 strain ATCC 43888 were created following the guidelines from the CLSI [33]. The bacterial concentration was adjusted to a final inoculum of 1.5 × 10^6^ cfu/mL and then exposed to various antibiotic concentrations ranging from 1× to 4× MIC. Control growth curves were established using MHB without any antibiotics. Bacterial counts were conducted at various intervals: 0, 1, 2, 4, 8, 12, and 24 h of culturing, after which they were incubated for 24 h at 37 °C on LB plates [34].

### 4.5. PD Modeling

The study investigated the PD relationship between the concentration of an antibiotic and the corresponding growth and death rates of bacteria [18]. A model was proposed to depict the net growth rate (ψ) of a bacterial population when exposed to a particular antibiotic concentration (a). This net growth rate is a function of various factors. In our model, the maximal bacterial growth rate is denoted as ψ_max_, while the bacterial death rate at a given antibiotic concentration is represented by μ (a), following a Hill function.

Essential parameters of this model include E_max_, indicating the maximum death rate induced by the antibiotic, and EC_50_, representing the antibiotic concentration at which the death rate is half of E_max_. The Hill coefficient, κ, is another critical parameter that describes the steepness of the curve relating μ to a, typically showing a sigmoidal relationship.

**Figure 6:**
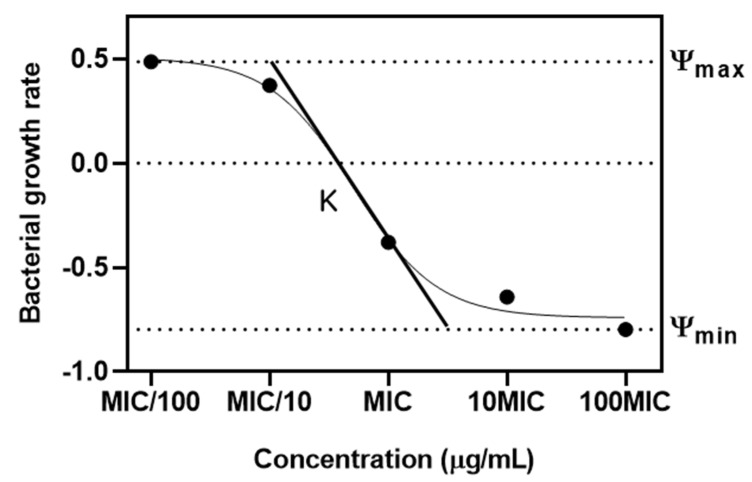
Pharmacodynamic model of relationship between antibiotic concentration and bacterial growth. Ψ represents the net growth rate of the bacteria. ψ _max_ and ψ _min_ are maximal bacterial growth rate and minimal bacterial growth rate. κ defined as Hill coefficient is the steepness of the curve.

To quantify these rates, such as ψ (a), ψ_max_, μ (a), and E_max_, we evaluated the hourly logarithmic changes (base 10) in bacterial density. Furthermore, zMIC is defined as the PD MIC at which no bacterial growth is observed, meaning ψ (zMIC) is zero. Figure 6 in our study illustrates how these parameters affect the relationship between antibiotic concentration and bacterial growth rate.

(1) $$\psi \left(a\right)=\psi_{max}-\mu (a)$$

(2) $$\mu (a)=E_{max}\frac{{\left(\frac{a}{{EC}_{50}}\right)}^{\kappa }}{1+{\left(\frac{a}{{EC}_{50}}\right)}^{\kappa }}$$

(3) $$\psi \left(a\right)=\psi_{max}-\frac{(\psi_{max}-\psi_{min}){\left(\frac{a}{zMIC}\right)}^{\kappa }}{{\left(\frac{a}{zMIC}\right)}^{\kappa }-\psi_{min}/\psi_{max}}$$

### 4.6. Monte Carlo Simulation

In this approach, the rate of bacterial growth, derived from the data of time–kill curves, underwent a transformation into logarithmic scale. Following this, a detailed model was established to link these logarithmic values with their corresponding responses. The process of generating subsequent data points involved adding a fixed increment of 0.1 to each value. To incorporate randomness and variability in the data, a Gaussian scatter model was applied, characterized by a standard deviation of 0.1.

### 4.7. Statistical Analysis

The data were presented as mean values accompanied by standard deviations. For statistical analysis, ANOVA (analysis of variance) was utilized, executed via the GraphPad Prism software (version 8.0.1, based in La Jolla, CA, USA). A *p*-value below 0.05 was considered statistically significant.

## 5. Conclusions

In summary, facing the growing menace of antimicrobial-resistant *E. coli*, the integration of novel experimental methodologies and mathematical modeling will play a crucial role in guiding future research and developing new treatment approaches. Although the journey forward is filled with obstacles, employing thorough and scientific methods such as those demonstrated in this study equips us more effectively to tackle the intricate issues surrounding antimicrobial resistance.

## Figures and Tables

**Table 1:** Minimum inhibitory concentration and minimum bactericidal concentration of 5 antibiotics against *E. coli*.

Antibiotics	MIC	MBC	MBC/MIC
SMN	2	4	2
KMN	1	2	2
GMN	0.25	1	4
TMN	0.5	1	2
AKN	0.25	1	4

Streptomycin, SMN; kanamycin, KMN; gentamicin, GMN; tobramycin, TMN; amikacin, AKN.

**Table 2:** Parameter estimates based on observed bacterial growth rate (n = 3).

Antibiotics	ψ_max_ (95% CI)	ψ_min_ (95% CI)	Hill Coefficient (95% CI)	EC_50_ (95% CI)	R^2^ (95% CI)
SMN	0.5651 (0.4419 to 0.7845)	−0.8166 (−1.028 to −0.6976)	−0.7631 (−1.244 to −0.4600)	2.996 (1.781 to 5.250)	0.9842
KMN	0.7290 (0.5889 to 1.022)	−0.9728 (−1.213 to −0.8488)	−0.5324 (−0.7153 to −0.3553)	1.374 (0.8118 to 2.202)	0.9938
GMN	0.5240 (0.4212 to 0.6701)	−0.7685 (−0.9204 to −0.6699)	−0.9266 (−1.694 to −0.5834)	0.7419 (0.4936 to 1.174)	0.9854
TMN	0.3745 (0.2323 to 0.5583)	−0.8218 (−0.9516 to −0.7168)	−1.323 (−2.068 to −0.7063)	0.2389 (0.1355 to 0.3037)	0.9682
AKN	0.5249 (0.3646 to 1.001)	−1.141 (−2.027 to −0.9152)	−0.5799 (−0.9459 to −0.2582)	1.5290 (0.7134 to 7.856)	0.9794

ψ_max_ is maximal bacterial growth rate; ψ_min_ is maximal bacterial growth rate; EC_50_ is the value to produce 50% of the maximal antibacterial effect.

**Table 3:** Parameter estimates of antibiotics through Monte Carlo.

	ψ_max_	ψ_min_	ψ_max_ − ψ_min_	ψ_min_/ψ_max_	Hill Coefficient	zMIC
SMN	0.46 ± 0.05	–0.92 ± 0.13	1.38 ± 0.08	–1.50 ± 0.61	–0.65 ± 0.12	1.22 ± 0.19
KMN	0.90 ± 0.13	–0.99 ± 0.12	1.89 ± 0.01	–1.90 ± 0.08	–0.50 ± 0.09	0.89 ± 0.52
GMN	0.46 ± 0.05	–0.76 ± 0.04	1.22 ± 0.01	–1.60 ± 0.25	–1.00 ± 0.16	0.21 ± 0.02
TMN	0.36 ± 0.04	–0.83 ± 0.02	1.19 ± 0.02	–1.43 ± 1.00	–1.56 ± 0.20	0.32 ± 0.15
AKN	0.55 ± 0.13	–0.99 ± 0.15	1.54 ± 0.01	–1.55 ± 0.08	–0.53 ± 0.14	0.13 ± 0.02

zMIC, pharmacodynamic MIC.

## Data Availability

Data is contained within the article.
